# A Review in Management of Testicular Cancer: Single Center Review

**DOI:** 10.4021/wjon258w

**Published:** 2011-06-08

**Authors:** Ammar Hameed, Bob White, Frank Chinegwundoh, Ali Thwaini, Ajay Pahuja

**Affiliations:** a Department of Urology, Addenbrookes Hospital, Cambridge, UK; b Teesside University, UK; c Department of Urology, St Bartholomew’s Hospital, London, UK; d Department of Urology, Belfast City Hospital, North Ireland, UK

**Keywords:** Testicular cancer, Age-adjusted incidence, Management, Orchidectomy, IGCCCG

## Abstract

**Background:**

Testicular cancer is one of the few solid cancers that can be cured even when it is metastasized with overall survival rate of more than 90%. The aim of this study was to establish the age adjusted incidence of testicular cancer and to critically assess the management of testicular tumor.

**Methods:**

This is a quantitative retrospective study utilizing a review of clinical notes for patients who underwent testicular orchidectomy. The number of cancer cases, types of pathology and cancer staging were examined.

**Results:**

There is no substantial difference between the crude and the age-standardized incidence, moreover no difference from the reported crude incidence by the Scottish intercollegiate guidelines. We found 55.1% of seminoma, 14.28% of non-seminoma and 30.61% of combined (seminoma and non-seminoma), and stage I disease in 61.22% of cases, stage II in 36.73% of cases, and stage IV in 2.04% of cases. Most of the cancers were in the age group 20 - 50 with the majority (48.97%) in the age group 31 - 40. About 42.85% of cases were identified with high tumor markers; higher percentage of seminoma at stage II (40.74%).

**Conclusions:**

There is no substantial difference between the crude and the age-standardized incidence, moreover no difference from the reported crude incidence. Most of the cancers were in the age group 20 - 50 with the majority (48.97%) in the age group 31 - 40. Only 25% of seminomas had elevated tumor markers. Moreover, it is important to re-enforce strict adaptation to the IGCCCG prognostic factor-based classifications.

## Introduction

Testicular cancer is the most prevalent cancer among young men aged 15 to 35 years, with crude incidence of 7.52 cases per 100,000 of the population. Though rare, it is one of the most curable solid cancers and serves as a model amongst malignancies treated with various approaches. The mortality rate of testicular cancer has decreased dramatically from 50% before the 1970s to almost less than 5% in recent years [1].

Overall, the highest incidence is noted in young adult males, making this neoplasm the most common type of solid tumor in men aged 20 to 34 years and the second most common tumor in men aged 35 to 40 years in the United States and Great Britain [2].

The etiology of testicular cancer is largely unknown, and there is little explanation for the changing incidence. The incidence of testicular germ cell cancer rose in all age groups from 2 - 3/100,000 to 5 - 6/100,000 in the late 1980s. Over 70% of all cases occur in men under 40 years old, and in this age group the increase in incidence is most prominent, from 4 - 10/100,000 over 1961 - 1988 [3]. In the UK the incidence is 7/100,000 and is the commonest form of solid cancer in men aged 18 to 35 years [4].

### Age distribution

Fifty percent of seminomas are seen in men in their thirties. Seminoma rarely, if ever, occurs in the adolescent or infant population, but it may occur in patients older than 60 years [5].

It is well known that embryonal carcinoma and terato-carcinoma occur predominantly between 25 - 35 years. Chorio-carcinoma (1 - 2% of all germ cell tumors) occurs more often in the 20-30-year age group.

### Tumor markers

Serum tumor markers are very important contributing factors in the diagnosis and management of testicular tumors, including AFP (produced by yolk sac cells) and B-HCG (expression of trophoblasts).

The rate of tumor marker decline subsequent to treatment represents an important prognostic index; they increase in 51% of cases. In non-seminomatous germ cell tumors, AFP increases in 50 - 70% of cases and B-HCG in 40 - 60% of cases. On the other hand, 30% of seminomatous germ cell tumors have raised B-HCG [6].

### Clinical staging

In addition to its prognostic value, clinical staging plays a key role in decision-making with respect to appropriate treatment. The fact that more treatment protocols are established for low-risk tumors makes accurate clinical staging vital.

In patients with clinical stage I disease, 10 - 15% harbour undetected nodal metastasis and another 5 - 10% relapse after surgery in extra-nodal sites [7]. These figures underscore the need for patients on surveillance protocols to adhere diligently to the protocol. For purpose of simplicity, staging is divided into seminoma and non-seminomatous tumors. Where pure seminomas are usually staged by clinical means, surgical techniques such as retroperitoneal lymph node dissection are used for non-seminomatous tumors [8].

### Study objective

To establish the age-adjusted incidence of testicular cancer as well as the number of cancer cases with pathology type and clinical staging.

## Methods

This is a quantitative retrospective study utilizing a review of clinical notes for 109 patients who underwent testicular orchidectomy. The number of cancer cases, types of pathology and cancer staging were examined.

### Data analysis

A descriptive statistical method is employed in data analysis in order to measure the percentages, means and variances.

## Results

### Tumor histology

**Figure 1:**
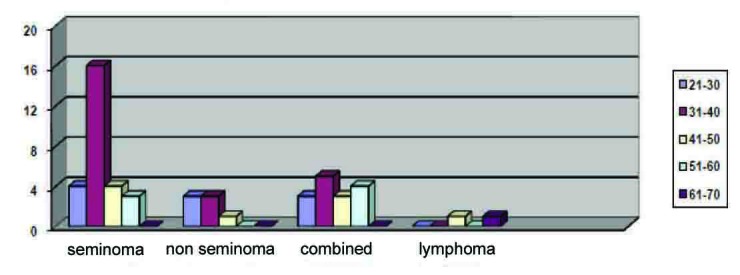
Tumor histology/age distribution.

Out of the 98 cases identified, 49 (50%) cases were reported as cancer and 2 (2.04%) as lymphoma. Of the cancer patients, 27 (55.1%) cases were seminomas, 7 (14.28%) were non-seminoma and 15 (30.61%) as MCT (seminoma and non-seminoma). (Fig. 1)

### Cancer incidence

The age-standardized incidence rate reflects a review of the individual age-specific rates employing a standard population, representing the incidence that could be observed if the age structure of the sample population corresponded to a standard population. The age-standardized incidence rate is defined as the number of new cases per 100,000 person-years.

As shown in Table 1, the crude rate is 48/700397 = 6.99/100,000 person-years, and the age-standardized rate is 7.3/100,000 person-years. The age-standardized rate is only marginally higher than the crude rate because the standard population is on average similar to the sample population.

**Table 1:** Testicular Tumor Incidence (Age Standardized) (2005 - 2009)

Generation	No. of cases	Person-years at risk	Age-specific incidence (per 10^5^ years)	Standard European population	Expected cases in standard population
I	d_i_	y_i_	10^5^ (d_i_ /y_i_)	w_i_	d^i^ w^i^/y^i^
0 - 4	0	37,610	0.00	8000	0.00
5 - 9	0	43,094	0.00	7000	0.00
10 - 14	0	49,357	0.00	7000	0.00
15 - 19	0	47,870	0.00	7000	0.00
20 - 24	4	39,504	10.126	7000	0.71
25 - 29	5	35,090	14.249	7000	1.14
30 - 34	7	44,878	15.598	7000	0.99
35 - 39	11	52,644	20.895	7000	1.46
40 - 44	10	53,373	18.736	7000	1.31
45 - 49	4	49,851	8.024	7000	0.56
50 - 54	8	49,390	16.198	7000	1.13
55 - 59	0	50,324	0.00	6000	0.00
60 - 64	0	40,691	0.00	5000	0.00
65 - 69	0	36,419	0.00	4000	0.00
70 - 74	0	29,011	0.00	3000	0.00
75 - 79	0	20,835	0.00	2000	0.00
80 - 84	0	13,040	0.00	1000	0.00
85+	0	7,416	0.00	1000	0.00
Total	49	700,397	6.99	100 000	7.3

Incidence = All new cases in 2005 - 2009/total population (2005-2009). *Scandinavian (“European”) standard is used.

### Tumor markers

Among cases with pre-orchidectomy tumor markers, 27 had normal tumor markers and 21 (42.85%) had raised tumor markers. Out of the latter, AFP was raised in 7 (14.28%) cases; B-HCG in 8 (16.32%) cases; both in 6 (12.24%) cases; and 1 case not reported.

In seminoma group, 7 (25.92%) cases had raised B-HCG, 19 (70.37%) normal tumor markers, and 1 (3.71%) case not reported. In the non-seminoma group, 2 (28.57%) had raised AFP, 1 (14.29%) raised B-HCG, 2 (28.57%) raised both markers and 2 (28.57%) cases normal. Finally, 15 cases were MCT, with raised AFP in 5 (33%), both tumor makers rose in 4 (26.6%) and normal tumor markers in 6 (40%).

### Tumor/stage managements

Table 2 demonstrates the tumor histology and staging.

**Table 2:** Tumor Histology and Staging

Tumor type	Stage	Number = 49	Percentage
Seminoma	I	16	59%
Seminoma	II	11	40.74%
Non seminoma	I	5	71.42%
Non seminoma	II	2	28.58%
Mixed tumor	I	9	60%
Mixed tumor	II	5	33.34%
Mixed tumor	IV	1	6.76%

Seminoma stage (I): 16 (32.65%) cases; 12 (75%) had radiotherapy, 3 (18.75%) had chemotherapy, and 1 (6.25%) was surveilled.

Seminoma stage (II): 11 (22.44%) cases; 3 (27.27%) had radiotherapy and 8 (72.73%) had chemotherapy.

Non-seminoma stage (I): All of the 5 (10.2%) were kept under surveillance.

Non-seminoma stage (II): All of the 2 (4.08%) cases had chemotherapy.

Mixed (seminoma/non-seminoma) Stage (I): 9 (18.36%) cases; 2 (22.22%) had chemotherapy and 7 (77.78%) had surveillance.

Mixed (seminoma/non-seminoma) Stage (II): All 5 (10.2%) cases received chemotherapy.

Mixed (seminoma/non-seminoma) Stage (IV): 1 (2.04%) case had combined chemotherapy and radiotherapy.

## Discussion

Germ-cell tumor (GCT) is the most common cancer in young men aged 15 to 35 years, and is therefore an extremely important cancer to treat. It is potentially curable by radiotherapy and chemotherapy, even in cases of widespread metastases, elevated tumor markers and other adverse prognostic features.

### Incidence of testicular cancer

Comparisons of crude age-specific incidence rates for a specific time and between different populations can lead to unrealistic outcomes, mainly because age comparisons may differ between populations. The use of direct age standardization has become an indispensable tool for academic comparison in relation to age-specific epidemiological rates between populations with different age compositions. The dominant method currently in operation is the direct age-standardization of rates in an arbitrary standard population [9, 10].

There is no substantial difference between the crude and the age-standardized incidences. The overall impression is that testicular tumor incidence in the population studied has been stable. Yet, taking these results in general terms does reflect either a reduction in the incidence or under-diagnosis; further studies will be needed to ascertain that fact.

### Age distribution

Seminoma: 4 (14.81%) cases were found in the 21 - 30 age group; 16 (59.25%) in the 31 - 40 age group; 4 (14.81%) in the 41 - 50 age group; and 3 (11.11%) cases in the 51 - 60 age group.

Of the 7 (14.28%) non-seminoma cases reported, 3 (42.85%) cases were in the 21 - 30 age group; 3 (42.85%) cases in the 31 - 40 age group; and 1 (14.28%) in the 41 - 50 age group.

In addition, there were 15 (30.61%) cases of MCT: 3 (20%) cases in the 21 - 30 age group; 5 (33.33%) cases in the 31 - 40 age group; 3 (20%) cases in the 41 - 50 age group; and 4 (26.67%) cases in the 51 - 60 age group. There is a slight difference in age-distribution with MCT-more than 30% of them tend to be in the older age group, yet most of the cases (53.33%) were in the 21 - 40 age group.

### Tumor stages

In large population-based patient series, 75 - 80% had seminoma and 55% had NSGCT at stage I; approximately 15 - 20% of seminoma patients were categorized as stage II, compared to around 40 - 50% of NSGCT, bearing in mind that up to 30% of NSGCT patients with clinical stage I disease have sub-clinical metastases and will reflect a higherstage of the disease [5, 11].

Our study results demonstrate a higher percentage of seminoma in general compared to non-seminoma; more seminoma in stage II; the non-seminoma group had a higher percentage of stage I with lower cancer levels in stage II, this may have an important impact on treatment options, survival and cure rate.

MCTs account for approximately 30 - 50% of testicular tumors. Few studies reflect these percentages, although they have an impact on treatment options [12]; MCTs comprise 30% of testicular cancer, with 60% in stage I and 33% in stage II. Only one case was at stage IV (7%); therefore most of the cancer cases (93%) were at stage I and II.

### Treatment

#### Seminoma disease

##### 1) Stage I seminoma disease

The level of the tumor markers should normalize within a reasonable time (half-life time for HCG: 2 - 3 days; half-life time for AFP: 5 - 6 days). Prolonged half-life times after orchidectomy suggest the presence of residual AFP or HCG producing cells, not compatible with stage I disease [5]. Usually, radical orchidectomy is the first step in the treatment of testicular cancer before any other treatment modalities.

###### a) Surveillance

This method has been used as a research protocol in the management of stage I seminoma. The risk of delayed relapse spreads over the first five years, usually occurring in the para-aortic nodes. Seminoma treatment is necessary in only 20% of cases, namely those with microscopic disease, thus treatment of all patients with stage I disease means over-treatment in 80% of cases. Surveillance is based on the clinical experience that the post-orchidectomy treatment is usually salvaged by either radiotherapy or chemotherapy if they relapse.

Daugaard et al, (2003) found a relapse rate of 17% of patients, 87% of these within 2 years. Warde et al, (1993) found a 5-year progression-free rate of 82%. In our study seminoma stage I was found in 16 cases, one case had surveillance with a clear intention to avoid any delayed relapse. He had a localised tumor, no local extension, normal post orchidectomy tumor markers and negative computerised tomography (CT) [13, 14].

###### b) Radiotherapy

Seminomatous tumors are sensitive to radiation with cure rate of 95%. The first lymph-node metastases usually occur in the para-aortic region with the exception of prior inguinal or scrotal surgery. When the para-aortic lymph nodes are tumor free, it is extremely unlikely that iliac lymph-node metastases are found [15]. About 3 - 5% of the irradiated patients may relapse, though most of them can be cured with salvage chemotherapy up to 99% [16]. In our study 12 patients with sage I seminoma had radiotherapy. Four cases had positive lymph nodes; all of them had normal tumor markers after radical orchidectomy.

###### c) Chemotherapy

Seminomas are highly sensitive to chemotherapy (cisplatin-based). A multicenter trial [17] randomized patients with stage I seminoma to either single-agent chemotherapy or surveillance and observed a relapse rate of 3.3% after 52 months in the chemotherapy group. A large randomized control trial [18] randomizing patients between radiotherapy and single course of chemotherapy, median follow-up of 4 years, confirmed no significant difference in relapse and survival. With chemotherapy, the patient’s treatment period is shortened, with less acute subjective toxicity and reduced risk of late effects compared with radiotherapy. In this study 3 cases had chemotherapy and showed good tolerability and few side effects.

##### 2) Stage II seminoma disease

Fifteen percent of patients fall into this category, it can be further subdivided into subgroups reflecting the prognostic significance of bulk retroperitoneal disease, but in our study we will consider the management of stage II in general.

The data on the specific results and relapse rate of radiation therapy in the management of stage II varies. The Royal Marsden Hospital reported a conflicting outcomes in randomized studies with a relapse rate of 0 - 11%, it may well be that the selection criteria played a major role in the observed difference, a relapse-free survival after 6 years for stage II of 89 - 95% with an overall survival is almost 100% [19].

With a survival rate reaching from 85% to 100% chemotherapy is the preferred initial therapy; additionally it is difficult to deliver chemotherapy for those relapsed after radiotherapy. When larger retroperitoneal disease exits chemotherapy is preferred since serial trails indicate radiotherapy will lead to high relapse rate (35%), in addition to large irradiation area, this will add more toxic effects [20]. In our study seminoma stage II was reported in 11 (40.75%) cases, 3 (27.27%) had radiotherapy and 8 (72.73%) had chemotherapy. More patients are being treated with chemotherapy for three main advantages: reduces post treatment relapse, less systemic effect and not useful after radiotherapy.

#### Non-seminoma germ cell carcinoma

##### 1) Stage I non-seminoma disease

###### a) Surveillance

Surveillance is very popular, has less treatment morbidity and has the capacity to resort to chemotherapy on relapse. Studies showed a consistent relapse rate of around 30% and certain studies like the MRC/EORTC have enabled subgroups of patients to be identified and categorized as high risk of relapse on surveillance. Blood vessel and/or lymphatic invasion represent the main histological feature for relapse with a risk of approximately 40%; 80% of relapses occurr during the first 12 months of follow up, 12% during the second year and 6% during the third year [21]. In around 35% of those relapses, tumor markers maintain normal levels. The retroperitoneum is the site of around 60% of relapses and large volume disease occurs in 11% of them [22]. Based on the overall cancer-specific survival data, surveillance within an experienced surveillance scheme may be offered to patients with this stage, ensuring compliance and clear information regarding recurrence rate as well as the salvage treatment. Our study identified 5 (71.42%) cases with stage I non-seminoma and all had surveillance. In all cases no vascular or lymphatic invasions were identified histologically and no lymph node involvement on CT scan.

###### b) Chemotherapy

Patients of this stage and with high-risk features should be considered for adjuvant chemotherapy. Several randomized control studies have indicated two cycles of chemotherapy regime as primary treatment for high-risk patients (those with 50% risk of relapse). The relapse rate for chemotherapy is only 2.7%, with very little long-term toxicity with effect on fertility or sexual activity. It is important to be aware of the slow-growing and late retroperitoneal chemo-resistant cancer relapse [23]. Patients with low risk disease who are unable or unwilling to follow a policy of surveillance may be treated with two courses of chemotherapy. Although in our study most of the patients had surveillance due to low risk, it seems that the choice of chemotherapy is based on the risk factors and the patients’ preferences.

##### 2) Stage II non-seminoma disease

The cancer cure rate with chemotherapy is 98%, these cancers spread by both lymphatic and blood vessel channels. Their prognosis and aggressiveness are related to tumor marker level and the anatomical extent of spread. The International Germ Cell Consensus Classification incorporated these features (the prognostic factor based staging) [24]. About 30% of patients will not achieve a complete remission after chemotherapy and will need a residual tumor resection [25].

The current study has identified two cease in stage II, both cases had chemotherapy with no reported complication. In one case vascular invasion was reported histologically with no lymph node involvement and normal tumor markers post orchidectomy while the other case suggested some insignificant lymph node in the pulmonary area with no localized (vascular or lymphatic) invasion. It is very clear that chemotherapy is a safe option in both cases as their tumor markers were not hugely elevated.

#### Mixed (seminoma and non-seminoma) cell disease

##### 1) Stage I mixed cell disease

It has an older mean age at presentation, the relapse rate is identical to those with non-seminoma; 80% relapse within the first year: 47% abdominal nodes, 17% lungs and 23% tumor marker rise [26].

Patients with no high risk features could be managed by surveillance following inguinal orchidectomy; adjuvant chemotherapy is offered to patients with high risk features (blood/lymphatic invasion) or if the patient is unable to comply surveillance policy [21]. In our study 7/9 cases had surveillance, 5 had no vascular/lymph node invasion (low risk). In 2 cases and despite vascular involvement and tumor marker (AFP) failed to normalize, they were enrolled in surveillance program, as part of clinical trial and they were unable to have chemotherapy. These two cases had tumor relapse with liver metastasis and went to have chemotherapy. There is a need for more aggressive treatment and highlight the importance of staging accuracy with the IGCCCG prognostic factors.

##### 2) Stage II mixed cell disease

The risk of relapse will be higher and the possibility of microscopic distant deposits is not uncommon; treatment is with chemotherapy to avoid relapse which can sometimes reach 30% and subsequent residual resection may be needed. The number of chemotherapy cycles will depend on the localised extension (vascular/lymphatic), the tumor markers and lymph node involvement, yet the presence of vascular invasion seems to be a very robust parameter [24]. In our results five patients at this stage had mixed features of localized invasion and lymph node involvement with good response of tumor markers post orchidectomy; all of them had chemotherapy.

#### Metastatic mixed cell disease

The primary treatment of choice is chemotherapy, number of cycles and duration vary between centers and toxicity levels; also it follows the IGCCCG risk classification.

Patients with ‘good prognosis’ received three-cycles of chemotherapy; delaying any chemotherapy cycle is justified only if fever with granulocytopenia or thrombocytopenia [27]. The ‘intermediate prognosis’ group has 80% 5-year survival rate [28]. Patients with a ‘poor prognosis’, standard treatment consists of 4-cycles of chemotherapy. The 5-year progression-free survival is between 45% and 50%. Patients with a slow marker decline may represent a prognostically inferior subgroup [29]. In our study only one patient was identified with both pulmonary and lymph nodes involvement and was treated with combined localised radiotherapy and chemotherapy. Both modalities were used as radiotherapy is beneficial to residual tumor, endorsed by the Scottish intercollegiate guidelines as a method of choice for post chemotherapy residual tumor.

### Conclusion

Our overall impression: there has been no increase in the incidence of testicular tumors in the population sample of this study as compared to previous rates. The age distributions for seminomas and non-seminomas are compatible with international published literature; however, mixed tumors were more among the older age groups.

Only 25% of seminomas had elevated tumor markers, while non-seminomas and mixed-cell carcinomas had low levels of elevated tumor markers. This highlights that tumor markers are not as high-yielding as anticipated. We found more seminoma cases compared to non-seminomas and a higher percentage of mixed cell tumors, which reflects the heterogeneity of testicular cancer in the study population. Moreover, it is important to re-enforce strict adaptation to the IGCCCG prognostic factor-based classifications.
